# Artificial mediators, protein carriers, and catalyst architecture in C1 bioconversion

**DOI:** 10.3389/fbioe.2026.1967754

**Published:** 2026-09-23

**Authors:** Sung Heuck Kang, Mohd Faheem Khan, Suk Min Kim

**Affiliations:** 1 Department of Biotechnology, The Catholic University of Korea, Bucheon-si, Republic of Korea; 2 UCD School of Agriculture and Food Science, University College Dublin, Dublin, Ireland

**Keywords:** C1 bioconversion, carbon monoxide dehydrogenase, catalyst architecture, electron carrier, ferredoxin, formate dehydrogenase, hydrogen, redox mediator

## Abstract

Biological conversion of one-carbon (C1) feedstocks frequently requires coupling oxidative and reductive reactions through an appropriate electron-transfer pathway. The combination of carbon monoxide dehydrogenase (CODH), which oxidizes CO to CO_2_, and formate dehydrogenase (FDH), which reduces CO_2_ to formate, serves as a representative model for examining this requirement. CODH–FDH coupling has been investigated in cell-free systems, whereas intracellular and distributed configurations provide emerging or prospective routes for extending this reaction across different biological environments. However, the relationship between electron carrier selection and system implementation is not one-to-one. Small artificial mediators such as viologens can connect physically separated enzymes or cells when they can access the relevant redox components, whereas protein carriers such as ferredoxins generally favor arrangements in which compatible partners share an accessible intracellular environment. When reactions are divided between cell populations, the connection may instead depend on a diffusible redox metabolite or an engineered conductive interface. Carrier mobility, accessibility across physical boundaries, partner recognition, and chemical stability constrain the range of system arrangements available for C1 bioconversion. Using CODH and FDH as a representative model, this Mini Review examines how electron-carrier properties and spatial organization shape C1 bioconversion architectures under process-relevant conditions.

## 1 Introduction

Biological conversion of one-carbon (C1) compounds provides routes for carbon recycling and fuel and chemical production from carbon monoxide, carbon dioxide, and formate (Hwang et al., 2020; Cowan et al., 2025). Many C1 pathways combine an oxidative reaction that generates reducing equivalents with a reductive reaction that produces the target compound (Schuchmann and Müller, 2013; Schwarz et al., 2020; Lim et al., 2022), and pathway performance depends on electron transfer between catalytic reactions that may occur in different proteins, cellular compartments, organisms, or process phases (Lim et al., 2022; Kim et al., 2026).

Electron carriers differ in molecular size, diffusibility, membrane accessibility, interaction specificity, and regeneration mechanism (Hattori et al., 2001; Kurisu et al., 2001; Reguera et al., 2005; Miyaji and Amao, 2020). Small soluble mediators can connect physically separated enzymes, whereas protein carriers generally require compatible intracellular partners. Reactions divided between cell populations need a molecule or interface that crosses or bypasses the cellular boundary (Summers et al., 2010; Rotaru et al., 2012; Kim et al., 2026). Redox potential, reaction rate, stability, and regeneration (Axley and Grahame, 1991; Harmer et al., 2023; Groh et al., 2024) remain important, but carrier mobility and partner recognition also determine which enzyme, cell, and interface arrangements are feasible.

Carbon monoxide dehydrogenase (CODH) and formate dehydrogenase (FDH) provide a useful model for examining these relationships. CODH oxidizes CO to CO_2_ (Jeoung and Dobbek, 2007), whereas reductive FDH converts CO_2_ to formate (Reda et al., 2008; Bassegoda et al., 2014). The difference between the CO_2_/CO and CO_2_/formate redox couples can provide a positive driving force for electron transfer under appropriate biochemical conditions (Schuchmann and Müller, 2013; Schwarz et al., 2020; Lim et al., 2022). However, thermodynamic feasibility alone does not establish functional coupling. Electron transfer also depends on carrier accessibility, enzyme-carrier compatibility, and the kinetics of the participating reactions. CODH and FDH provide the principal redox pair considered here, while related C1 and electron-transfer systems are used where direct CODH–FDH evidence is unavailable. Cell-free, intracellular, and multistrain systems show how carrier properties constrain the components that can be connected and the boundaries across which electrons can move (Figure 1A). Here, catalyst architecture denotes the organization of enzymes, cells, process phases, and electron-transfer components within the complete conversion system. Viologens and ferredoxins are treated as molecular and protein carriers, respectively. H_2_ and formate are diffusible redox metabolites, and conductive matrices and electrodes are transfer interfaces. These routes impose different requirements on feasible catalyst organization under process-relevant operating conditions.

**FIGURE 1:**
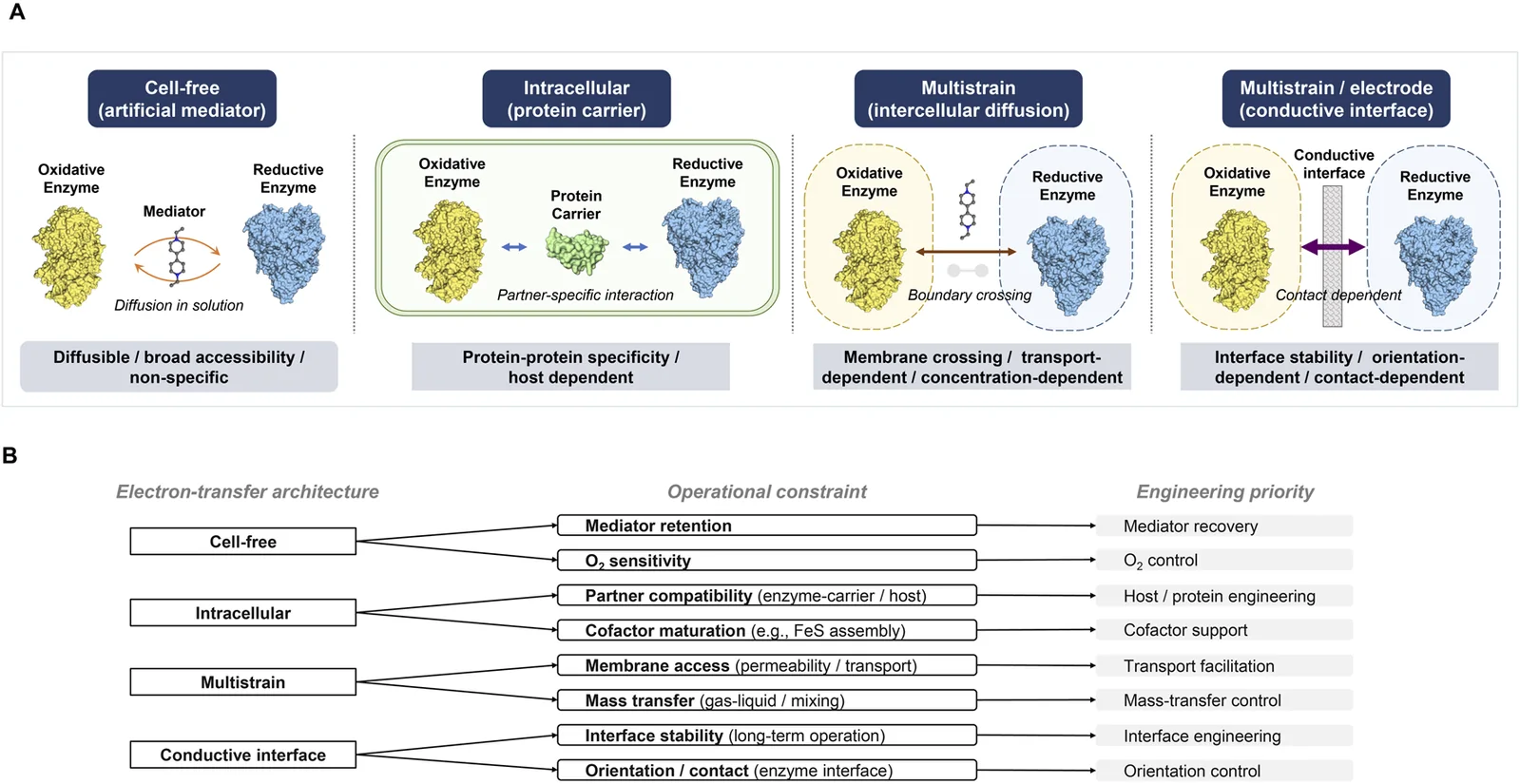
Electron-transfer architectures and the operational constraints they expose **(A)** Four electron-transfer architectures considered in this Review. Cell-free systems use a small diffusible artificial mediator to exchange electrons between physically separated enzymes. Intracellular systems rely on a protein carrier such as ferredoxin, which requires molecular recognition and remains within the cellular boundary. Diffusible multistrain systems use a mediator or H_2_ to transfer reducing equivalents through the extracellular medium between host populations. Conductive Intracellular transfer connects intracellular redox networks through an extracellular conductive pathway, whereas enzyme–electrode coupling depends on enzyme orientation and electronic coupling at a solid interface. Dashed ellipses represent cellular boundaries. **(B)** Different catalyst architectures expose different operational constraints. Cell-free systems expose mediator-retention and oxygen-related constraints, whereas intracellular systems depend on host compatibility and cofactor maturation. Diffusible multistrain systems can become limited by membrane access or gas–liquid mass transfer, while conductive and electrode-coupled systems introduce requirements for interface stability, orientation, and contact.

## 2 Cell-free systems with artificial mediators

### 2.1 Diffusible electron transfer in cell-free systems

When redox enzymes are removed from cells, they are separated from the native carriers that normally connect their reactions. Artificial mediators can replace this connection by exchanging electrons with the participating enzymes. Viologens are commonly used because they are small, soluble molecules that undergo reversible one-electron reactions and generally require less specific molecular recognition than protein carriers (Miyaji and Amao, 2020). Their small size and free diffusion allow enzymes to operate as separate components that can be dispersed, positioned, or replaced independently, and enzymes from different organisms can be combined through the same mediator. In the CODH–FDH model, viologen accepts electrons generated during CO oxidation and makes them available for CO_2_ reduction without direct contact between the two enzymes (Lim et al., 2022).

This flexibility supports modular catalyst organization. Enzyme amounts and ratios can be adjusted independently, while mediator concentration, pH, temperature, ionic composition, and substrate supply can be controlled directly, and the same carrier properties may support whole-cell or multistrain systems when the mediator can reach the relevant cellular redox components. Efficient coupling still requires biochemical compatibility. The mediator needs to reach all participating components and exchange electrons with them at rates compatible with the coupled reactions, and a mediator that reacts efficiently with one component but poorly with another may not sustain continuous transfer.

### 2.2 Limits of diffusible artificial mediators

The same diffusibility that allows an artificial mediator to connect separated enzymes also exposes it to the surrounding medium. A soluble mediator can react with unintended compounds or accumulate in one redox state when the coupled reactions are unbalanced. Soluble mediators also introduce retention and recycling requirements during repeated or continuous operation. These requirements do not eliminate this modularity, but they add operating requirements. Reduced viologen also reacts readily with molecular oxygen, transferring an electron to form superoxide while returning to its oxidized state (Krieger-Liszkay et al., 2011; Chen et al., 2018). This reaction competes with electron transfer to the intended component and may also damage enzymes or other reaction constituents. Direct enzyme inactivation needs to be distinguished from mediator oxidation because oxygen sensitivity varies among redox enzymes (Hartmann and Leimkühler, 2013; Basak et al., 2023). Anaerobic CODHs are one relevant example, as oxidation of their metal clusters can reduce catalytic activity. A soluble mediator may also be lost during product recovery or degraded through side reactions over repeated operation (Groh et al., 2024).

Mediator concentration is therefore a process variable rather than a simple means of increasing electron flux. Raising the mediator pool may accelerate transfer when carrier availability is limiting, but it can also increase nonspecific redox reactions, oxygen consumption, and downstream recovery load. The useful concentration range consequently depends on the complete coupled system rather than either enzyme alone.

## 3 Intracellular systems with protein carriers

### 3.1 Protein carriers and intracellular organization

Protein carriers such as ferredoxins differ from small artificial mediators in both size and interaction mode. They do not normally diffuse across cell membranes or move freely between separate cell populations. Electron transfer also requires transient molecular recognition between the carrier and its partner (Teshima et al., 2003; Bell et al., 2010). Their limited cellular mobility and requirement for molecular recognition favor arrangements in which the carrier and participating enzymes share an accessible intracellular environment (Ensign and Ludden, 1991). Ferredoxins contain FeS clusters that exchange electrons with redox enzymes. Productive transfer depends on the redox potentials of the components, the distance and orientation between their redox centers, and the ability of the carrier to approach, exchange an electron with, and dissociate from each partner (Mayoral et al., 2005). Redox compatibility alone is insufficient when unrelated proteins lack suitable interaction surfaces (Faro et al., 2002).

A proposed intracellular CODH–FDH system would require both enzymes to access a compatible protein carrier. Cellular metabolism can support carrier production and regeneration, avoiding an externally supplied mediator (Yacoby et al., 2011). Protein carriers do not imply one fixed arrangement within the cell. The carrier and its partners may remain freely distributed or may be organized through stable or transient associations (Bakkes et al., 2015). The relevant condition is continued access between compatible redox partners, not a single fixed intracellular format.

### 3.2 Carrier compatibility and host requirements

Colocation within a single cell does not ensure efficient electron transfer. A carrier that functions well with one enzyme may interact poorly with another, since differences in redox potential, binding surface, orientation, redox-center accessibility, and intracellular concentration can all affect transfer, particularly when the carrier and enzymes originate from different organisms (Bell et al., 2010). The carrier may also interact with native host proteins, diverting electrons from the pathway, and strong binding to one partner is not necessarily beneficial since productive transfer requires repeated binding and dissociation. A useful carrier combines productive interaction with the required partners and limited transfer to competing pathways.

Intracellular organization also links electron transfer to protein production and cofactor assembly. Ni–Fe CODHs require assembly of nickel- and iron-sulfur-containing clusters (Svetlitchnyi et al., 2001; Doukov et al., 2002), whereas many reductive FDHs depend on molybdenum- or tungsten-containing cofactors together with FeS centers and their own dedicated maturation machinery. High expression alone does not indicate a functional pathway when proteins remain insoluble or lack their cofactors, and functional production of an individual metalloenzyme supports component-level feasibility but does not establish CODH–FDH coupling through a shared ferredoxin. Demonstrating such a complete native-carrier CODH–FDH pathway would require active component production together with electron transfer and net coupled product formation, which has not yet been reported for this specific pairing.

## 4 Multistrain systems with intercellular electron transfer

### 4.1 Electron transfer across cell boundaries

The spatial limits of intracellular protein carriers become explicit when reactions are divided between cell populations. This division can place each reaction in a host suited to enzyme expression, cofactor assembly, or substrate use, but it interrupts electron transfer through a shared intracellular carrier. Ferredoxins generally remain within the cells in which they are produced. A ferredoxin reduced during CO oxidation in one population cannot directly reach FDH inside another. Multistrain coupling requires a transfer route that crosses or bypasses the cellular boundary.

Several routes can provide this connection. A small artificial mediator may move through the extracellular medium and exchange electrons with both populations without direct cellular contact. A diffusible reduced molecule, such as H_2_, may be produced by one population and consumed by another through the liquid and gas phases. A conductive pathway may instead connect cells through extracellular proteins, conductive structures, or an electrode, replacing free diffusion with direct contact. Despite their different chemistries, all three routes extend electron transfer beyond the range of an intracellular carrier, and in the CODH–FDH model, dividing the reactions between cells changes both the mode of electron transfer and the organization of the hosts.

### 4.2 Diffusible intercellular electron transfer

A diffusible artificial mediator extends the modularity of cell-free systems across cellular boundaries. Depending on the mediator and host, it may cross the membrane or exchange electrons with redox components near the cell surface. Its broad reactivity may connect populations that do not share a native transfer partner, allowing them to remain physically separate. Productive transfer still requires access to both populations. Membrane transport, uptake, export, intracellular accumulation, and chemical modification can differ between organisms. A mediator that enters one population more readily than the other may not support balanced transfer. The concentrations required for electron exchange may also affect growth or enzyme activity, while oxygen reactions, degradation, and loss from the culture can reduce long-term performance.

H_2_ provides a different form of diffusible intercellular transfer. One population can produce H_2_ through a hydrogenase, and another can consume it through its own hydrogenase and intracellular redox network. Each population retains its own protein carriers because H_2_ connects the two intracellular systems rather than replacing them. H_2_ imposes different transfer requirements from those of a soluble mediator. Interspecies H_2_ transfer between metabolically distinct partners is well established through the extracellular liquid phase (Rotaru et al., 2012), and in engineered gas-fed systems, it additionally proceeds through gas–liquid mass transfer, shaped by H_2_ partial pressure, gas flow, and interfacial area.

### 4.3 Conductive intercellular electron transfer

Conductive electron transfer connects separate intracellular redox systems through a biological or electrochemical interface instead of a freely diffusible reduced molecule. Some microorganisms exchange electrons with electrodes or other cells through extracellular redox proteins and conductive structures (Reguera et al., 2005; Summers et al., 2010). Productive transfer requires contact with the conductive pathway. This requirement affects population placement. Cells may need to form a structured community, attach to an electrode, or express membrane and extracellular proteins that connect intracellular metabolism to the external interface. Such systems have less spatial freedom than systems based on freely diffusible mediators.

Extracellular electron transfer is established in several microbial and bioelectrochemical systems, but application to a complete multistrain CODH–FDH pathway remains prospective. Electrons generated in one population would need to enter the external pathway and then reach the metabolism of a second population. Applied potential, electrode contact, biofilm formation, and competition at intracellular–extracellular boundaries could all affect performance. Diffusible mediators bridge separated cells but require recovery; H_2_ crosses cellular boundaries but is limited by mass transfer and thermodynamics; conductive routes avoid diffusible carriers but require effective interfacial contact.

## 5 Carrier properties and system organization

### 5.1 Carrier properties, system design, and evidence level

Carrier suitability begins with access. A carrier must reach each redox component, and protein-mediated transfer adds a further requirement for compatible molecular contact; once reactions are separated by a cellular or process-phase boundary, an additional transfer route becomes necessary (Table 1). These requirements are not equally demonstrated across the configurations compared here. Functional expression of an individual enzyme or carrier establishes only component-level feasibility, which is a distinct claim from evidence of electron transfer between components, and distinct again from net coupled product formation under the proposed operating conditions. Table 1 distinguishes the maturity of representative configurations from whether the supporting evidence comes directly from CODH–FDH configurations or from analogous systems. Even a demonstrated coupled pathway does not guarantee the same performance under process-relevant conditions, where gas supply, mediator recycling, and reactor-scale mass transfer introduce constraints absent from laboratory-scale validation.

**TABLE 1:** Representative electron-transfer configurations relevant to CODH–FDH C1 bioconversion.

System		Electron transfer		Representative case	Constraint/configuration maturity	Ref.
Type	Configuration	Carrier/route	Boundary/requirement			
Cell-free	Artificial-mediator coupling	MV, BV, EV	Bulk solution; enzyme-access dependent	Immobilized *Ch*CODH and *Me*FDH^a^ (real steel-mill off-gas, 10-L reactor)	Mediator handling, O_2_ reaction; demonstrated	Lee et al., 2024
	H_2_-driven enzyme coupling	H_2_ with MV or NAD^+^	Cell-free; H_2_ delivery	*Cn*Hyd and *Rc*FDH^b^ (MV outperformed NAD^+^, 18-h anaerobic stability)	Cofactor matching, H_2_ supply; demonstrated	Groh et al., 2024
Intracellular	Native protein relay	Dedicated FeS carrier	Intracellular; partner-specific	*Rr*CODH–*Rr*Hyd^b^ (22-kDa FeS protein required, ferredoxin/flavodoxin ineffective)	Partner specificity; demonstrated	Ensign and Ludden, 1991
	Engineered protein relay	FeS–FeS fusion	Intracellular; engineered proximity	Synthetic *To*CFOR^a^ (2.2 ± 0.2 μmol mg^–1^ min^–1^; purified enzyme)	Assembly/maturation; demonstrated	Lim et al., 2022
	Heterologous enzyme complex	Native FeS relay	Intracellular; maturation-dependent	*Aw*HDCR in *Escherichia coli* ^b^ (FeS reconstitution restored activity to 9.3 U mg^–1^)	FeS maturation; demonstrated	Leo et al., 2021
Multistrain	Diffusible-mediator coupling	EV	Cell envelope and extracellular phase	*Ch*CODH in *E. coli* and *Me*FDH^a^ (0.1 mM EV with permeabilization; FDH activity increased 7.9-fold)	Membrane accessibility; emerging	Kim et al., 2026
	Metabolite-mediated transfer	Formate/H_2_	Extracellular phase	*Tp* cells and methanogenic partner^b^ (Formate 5–9 μM; H_2_ 20–40 Pa)	Thermodynamic constraints; demonstrated	Hattori et al., 2001
	Conductive intercellular transfer	Conductive extracellular electron transfer	Cell–cell interface	Microbial nanowires and syntrophic aggregates^b^ (contact-dependent extracellular transfer)	Contact/interface organization; demonstrated	Reguera et al., 2005; Summers et al., 2010
Interface-coupled	Enzyme–electrode coupling	Direct electron transfer	Enzyme–solid interface	W-FDH on dye-sensitized TiO_2_ ^b^ (TOF 11 ± 1 s^–1^)	Orientation/interface stability; demonstrated	Miller et al., 2019

Configuration maturity refers to the experimental status of the representative configuration, irrespective of whether it has been demonstrated in CODH–FDH, coupling. Demonstrated denotes experimental validation of the stated configuration, Emerging denotes partial or early-stage validation, and Prospective denotes a configuration not yet demonstrated. Abbreviations: BV, benzyl viologen; CFOR, carbon monoxide–formate oxidoreductase; CODH, carbon monoxide dehydrogenase; EV, ethyl viologen; FDH, formate dehydrogenase; FeS, iron–sulfur; HDCR, hydrogen-dependent CO_2_ reductase; Hyd, hydrogenase; MV, methyl viologen; TOF, turnover frequency; *Aw*, *Acetobacterium woodii*; *Ch*, *Carboxydothermus hydrogenoformans*; *Cn*, *Cupriavidus necator*; *Me*, *Methylorubrum extorquens*; *Rc*, *Rhodobacter capsulatus*; *Rr*, *Rhodospirillum rubrum*; *To*, *Thermococcus onnurineus* NA1; *Tp*, *Thermacetogenium phaeum*.

^a^ Direct evidence from a CODH–FDH, configuration.

^b^ Analogous evidence from a related electron-transfer system.

Carrier performance should therefore be compared at the level at which electron transfer is actually required. A high rate measured with a purified enzyme does not establish effective transfer once a membrane, a competing redox network, or a second catalyst is introduced. This distinction is evident in formate dehydrogenase systems, where measured activity varies substantially with the route used to reach the catalytic center. Recent electrochemical measurements with *Desulfovibrio desulfuricans* formate dehydrogenase, for example, showed 7.5-fold and 5.8-fold increases in formate-oxidation activity with two different cytochrome mediators relative to unmediated conditions, showing that the electron-transfer route can change measured catalytic activity even when the enzyme is unchanged (Paul et al., 2025).

### 5.2 Operational implications

Diffusible artificial mediators support modular catalyst preparation but require mediator retention, oxygen control, and downstream removal as separate operations. Intracellular protein carriers avoid this external handling but link performance to host stability, cofactor maturation, and competing redox reactions, while distributed systems shift the requirement to whichever mediator, metabolite, or conductive interface connects the separated populations. Sustained coupling also requires carrier reduction and reoxidation to remain balanced; if the two half-reactions differ in capacity, the slower reaction constrains net flux.

The preferred carrier can also change with the intended mode of operation. In a batch reaction, mediator recovery may have little effect on short-term conversion, whereas continuous processing makes carrier retention and replacement part of the material balance. Whole-cell systems exchange this requirement for biological maintenance, and gas-mediated systems add mass-transfer constraints that are absent from soluble enzyme assays. For continuous operation, electron-transfer efficiency is only one criterion. Carrier location, regeneration or retention, and the material cost of repeated replacement can become equally relevant process variables.

## 6 Future perspectives

Several recent studies suggest that the boundary between artificial and native electron transfer is less fixed than a simple progression from viologens toward native carriers would imply. When a [NiFe]-hydrogenase was connected to formate dehydrogenase, methyl viologen supported a higher formate yield than the physiological cofactor NAD^+^, and the two enzymes retained activity together for 18 hours under anaerobic conditions (Groh et al., 2024). Physiological identity does not by itself guarantee a more effective coupling in an engineered system. A carrier’s suitability depends on the specific enzyme pair and reaction environment more than on whether it occurs in nature.

Protein-based coupling is also not confined to natural partner recognition. CODH and FDH lack a native interaction, yet an engineered iron-sulfur fusion protein supported CO-dependent formate production in both the purified complex and whole cells (Lim et al., 2022; Table 1). Physical fusion reduced the dependence on a pre-existing recognition interface by bringing the redox centers of two unrelated enzymes into close, productive proximity. Different catalyst architectures expose different operational constraints (Figure 1B). Heterologous production of the hydrogen-dependent CO_2_ reductase (HDCR) complex in *Escherichia coli* was initially limited by iron-sulfur (FeS) cluster maturation. *In vitro* FeS-cluster reconstitution substantially restored HDCR activity (Leo et al., 2021; Table 1). A whole-cell CODH–FDH cascade connected by ethyl viologen (EV) showed lower initial productivity than an immobilized-enzyme benchmark because the mediator’s access to the intracellular formate dehydrogenase was limited by the recipient strain’s envelope. Mild envelope permeabilization increased formate dehydrogenase activity 7.9-fold at 0.1 mM EV and improved cascade productivity under gas-fed reactor conditions, consistent with improved mediator access to the enzyme (Kim et al., 2026). This result supports membrane access as a constraint but does not establish carrier regeneration or half-reaction balance as the limiting step of the complete cascade.

Purified and immobilized cell-free systems have already been demonstrated beyond laboratory-scale batch reactions. A viologen-mediated CODH–FDH process converted untreated steel-mill off-gas into formate salts at molar-scale concentration in a pilot-relevant reactor (Lee et al., 2024; Table 1), while still carrying the mediator handling, oxygen exclusion, and enzyme immobilization requirements common to artificial-mediator systems. Electrode-coupled formate dehydrogenase offers a further route in which soluble carriers are removed altogether. A tungsten-dependent formate dehydrogenase adsorbed on dye-sensitized TiO_2_ reduced CO_2_ to formate under illumination without an added mediator (Miller et al., 2019; Table 1). This direct enzyme-electrode wiring differs mechanistically from the cell-to-cell conductive transfer discussed above, since it depends on stable enzyme orientation at a solid interface instead of contact between cell populations. Comparing these routes requires matched conditions, sustained-operation testing, and evaluation of architecture-specific constraints.

## 7 Conclusion

Electron carriers should not be evaluated only through redox potential or reaction rate with a single enzyme. Carrier mobility, partner recognition, and access across physical boundaries can restrict which catalyst arrangements are workable even when electron-transfer kinetics are favorable. Diffusible mediators permit modular organization but introduce retention and oxygen-related constraints, whereas protein carriers link transfer to partner compatibility and cofactor maturation. Dividing reactions between cell populations shifts the problem to the boundary between them. Electron carriers therefore constrain, without fully determining, how catalysts, cells, and transfer interfaces can be organized. Across the systems considered here, the more useful question is not which carrier is intrinsically preferable, but which boundary electrons must cross and what limits transfer at that boundary. Carrier selection is best considered after the required electron-transfer boundary has been defined. Once the locations of oxidation and reduction, the relevant cellular compartments, and the operating phase are established, candidate carriers can be evaluated against the specific requirements for access, recognition, regeneration, and retention imposed by that arrangement.
